# Type 1 Diabetes Modifies Brain Activation in Young Patients While Performing Visuospatial Working Memory Tasks

**DOI:** 10.1155/2015/703512

**Published:** 2015-07-22

**Authors:** Geisa B. Gallardo-Moreno, Andrés A. González-Garrido, Esteban Gudayol-Ferré, Joan Guàrdia-Olmos

**Affiliations:** ^1^Instituto de Neurociencias, Universidad de Guadalajara, Francisco de Quevedo 180, Colonia Arcos Vallarta, 44130 Guadalajara, JAL, Mexico; ^2^Facultad de Psicología, Universidad Michoacana de San Nicolás de Hidalgo, Francisco Villa 450, 58120 Morelia, MICH, Mexico; ^3^Facultat de Psicologia, Universitat de Barcelona, Institut de Recerca en Cervell, Cognició i Conducta (IR3C), Passeig de la Vall d'Hebron 171, 08035 Barcelona, Spain

## Abstract

In recent years, increasing attention has been paid to the effects of Type 1 Diabetes (T1D) on cognitive functions. T1D onset usually occurs during childhood, so it is possible that the brain could be affected during neurodevelopment. We selected young patients of normal intelligence with T1D onset during neurodevelopment, no complications from diabetes, and adequate glycemic control. The purpose of this study was to compare the neural BOLD activation pattern in a group of patients with T1D *versus* healthy control subjects while performing a visuospatial working memory task. Sixteen patients and 16 matched healthy control subjects participated. There was no significant statistical difference in behavioral performance between the groups, but, in accordance with our hypothesis, results showed distinct brain activation patterns. Control subjects presented the expected activations related to the task, whereas the patients had greater activation in the prefrontal inferior cortex, basal ganglia, posterior cerebellum, and substantia nigra. These different patterns could be due to compensation mechanisms that allow them to maintain a behavioral performance similar to that of control subjects.

## 1. Introduction

The devastating effects of Type 1 Diabetes (T1D) on the retinal, renal, cardiovascular, and peripheral nervous systems are widely acknowledged, but in recent years increasing attention has been given to the effect that this disease has on cognitive functions. Studies have reported that patients—including children—with T1D show cognitive deficiencies on a variety of neuropsychological tests when compared to healthy subjects [1, 2]; however, neuropsychological studies have been inconsistent with respect to the cognitive domains affected and the severity of the cognitive deterioration reported. This heterogeneity may be due to differences in the characteristics of the patients studied and the psychometric tests used [3, 4]. On the other hand, studies suggest that when the age of onset ranges from 5 to 7 years, children have a higher risk of cognitive dysfunction and are more likely to achieve poorer scores on neuropsychological tests [4–7].

Despite these efforts, the mechanisms underlying cognitive dysfunction in T1D are still poorly understood. The main issues to be resolved are (a) whether these mechanisms are related to specific changes in the brain [8] and/or (b) if there is any particular vulnerability effect on the developing brain that is associated with the disease evolution during childhood [9].

T1D is commonly diagnosed in childhood and adolescence, periods when the developing brain is undergoing dynamic changes such as myelination and synapse modification [10]. Therefore, abnormal glycemic variability can affect brain glucose metabolism and lead to neurocognitive deficits [4, 11]. In this regard, Ferguson and colleagues [6] reported that patients with early disease onset show structural brain abnormalities more frequently, suggesting that this could reflect suboptimal brain development. In fact, it has been hypothesized that young children are especially vulnerable to brain insults resulting from episodes of chronic hyperglycemia, hypoglycemia, and acute hypoglycemic complications that may adversely affect brain anatomy, brain metabolism, and brain function [2, 9, 12, 13].

There is general agreement on the notion that T1D seems to play a negative role in children's cognitive development which can still be detected in adulthood [1]. Moreover, one study suggested that children and adults show a similar pattern of cognitive performance [14]. Those authors assume that cognitive dysfunction occurs within the first two years after disease onset. In this regard, a review [13] also suggested that subtle brain structure abnormalities are detectable in patients shortly after diagnosis. This, however, is not always the case, as many T1D patients seem to lead normal lives with no evident cognitive difficulties. Also, it is possible to find young patients who have attained high levels of education and perform challenging jobs. Therefore, it is important to be sure not to assume that subtle brain abnormalities necessarily imply cognitive deficits.

Based on the documented negative effects of T1D on the central nervous system, one could infer that these patients are not exempt from future cognitive dysfunction or altered brain function. As a matter of fact, some studies have provided evidence that alterations may occur later in life as the disease progresses and this may be associated with the development of other diabetic complications [15, 16] that contribute to a slow and gradual deterioration of cognitive functions [17]. This notion leads to questioning whether early clinical emergence of T1D might subtly disturb brain function resulting in an evolutionary stage in which abnormal neural processing could, at least temporarily, successfully meet daily cognitive demands.

It appears that T1D has a negative impact on brain function that begins to take effect shortly after diagnosis [13, 14], such that in cases of early onset it might alter neurodevelopment, as various authors have suggested [10, 18, 19]. In this context, brain disturbances in adults with early-onset T1D are likely to reflect adaptive changes during brain development, which would make the brain more vulnerable to later deleterious effects due to exposure to diabetes-related factors [1, 20, 21]. In fact, several neuroimaging studies do suggest that the deleterious effects of T1D on neuropsychological functioning are related to structural and functional abnormalities in the brain [8, 22–29]. Although these studies were specifically conducted to assess the effect of glycemic extremes on the brain, some authors consider that T1D has a negative impact on cognition regardless of glycemic control [3, 30, 31].

In our view, it is crucial to evaluate cognitive function in patients with early-onset T1D during euglycemia (i.e., not only under abnormal hypoglycemic or hyperglycemic conditions), particularly when there is no evidence of cognitive difficulties, since it is still unclear whether or not brain function is affected during this particular stage of illness evolution. Consequently, we aimed to explore neurofunctional activation in young T1D patients with normal intelligence and without diabetic complications* versus* healthy control participants during a visuospatial working memory task.

If brain metabolism has been altered in these patients, as has been suggested [17, 32], then we would expect that the blood oxygen level-dependent (BOLD) fMRI activation response could reflect differences in cognitive processing between the two groups, especially considering that working memory is one of the cognitive domains [4] in these patients [3, 23, 33] that has most often been shown to be affected, and is most frequently explored.

## 2. Materials and Methods

Participants were selected through an intentional sampling method based on inclusion criteria. They included 16 patients with diagnosed T1D and 16 healthy control subjects. All were right-handed and had normal IQs (*t*(16.051) = −3.79, *p* = .709, and *r* = 0.69) and a minimum of 9 years of schooling. Patients had experienced at least 4 years of disease evolution and the age of onset was during childhood or adolescence. Despite our efforts to have a more homogenous sample in terms of the time of disease evolution, this variable in our group of patients ranged from 4 to 18 years. Potential subjects who had a history of neurodevelopmental disorders, neurological or psychiatric illness, or complications due to T1D (such as retinopathy, nephropathy, or neuropathy) were not included. Other exclusion criteria were alcohol and/or drug abuse and any contraindication to performing fMRI evaluation. The demographic and clinical characteristics of all subjects are shown in Table 1. Patients and control participants were matched by gender, age, and educational level. As expected, the T1D patients showed marked variability in glucose levels. The averages of glycated hemoglobin (HbA_1c_) and fasting plasma glucose in our group of patients were slightly above control levels, as is commonly observed in this illness. Plasma glucose levels were measured immediately before the fMRI study to prevent any bias in cognitive performance due to extreme glucose levels. All fMRI studies were performed in the morning after the subjects had eaten breakfast, so higher levels of plasma glucose were expected in both groups.

Fourteen of the patients were self-administering injections of either rapid- or long-acting insulin, dose dependent on carbohydrate count. The other two patients were treated with insulin pump therapy. All of them frequently self-monitor their blood glucose level and have a special diet as part of their disease control regimen.

### 2.1. Experimental Protocol

The study was reviewed and approved by the Ethics Committee of the “Fray Antonio Alcalde” Hospital. All volunteers, or their parents (when underaged), gave their informed written consent for the study.

During screening, patients filled out a questionnaire and provided the following information: handedness, medical history including their most recent glycated hemoglobin and fasting plasma glucose levels, and current treatment or medications. Their personal medical records were also thoroughly reviewed. Control participants also completed a clinical interview to ensure they belonged to the healthy normoglycemic population.

Plasma glucose was measured before the fMRI session (Accu-Check Active glucometer). During the scanning process, a visuospatial working memory task was presented. The task stimuli were administered using E-Prime Studio v.2.0 (Psychology Software Tools, Inc., 2010). Images were projected through a NordicNeuroLab's VisualSystem device and responses were collected using a magnetic resonance-compatible, hand-held, 4-button response pad connected to the computer by an optical cable interface.

The visuospatial working memory task consisted of a pair of assignments administered through a block design. In the first task (condition A), subjects were shown a trial sequence of 3 or 4 white squares positioned pseudorandomly around a fixation point on a black background. After a fixed delay, a corresponding sequence of 3 or 4 red squares was shown that either resembled (direct, 50%) or differed from (50%) the previous stimulation order. Subjects were required to press one button if the two spatial sequences were identical or a second button if they were not.

**Figure 1:**
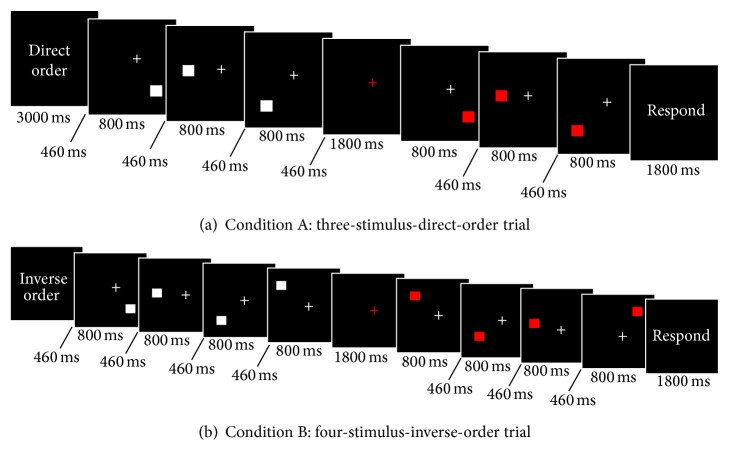
Schematic illustration of the experimental task. Each condition is presented in 4 activation blocks. A three-stimulus and a four-stimulus trial were presented in each activation block either in direct or inverse order according to the presented condition.

The assignment in condition B consisted in identifying, by pressing a button, whether the second sequence of red squares appeared in inverse order (inverse, 50%) or not (50%). If the latter sequence followed an order distinct from inverse, subjects were instructed to press a different button, but, in this condition, subjects were instructed to delay their responses until a warning signal with the command “Response” appeared.  Figure 1 shows the experimental flow chart. Responses and reaction times were recorded for each trial.

Prior to scanning, task instructions were properly explained to the subjects, who also performed several training trials with feedback in order to familiarize themselves with the tasks.

### 2.2. MRI Acquisition Methods

All images were acquired with a GE Excite HDxT 1.5 Tesla (General Electric Medical System, Milwaukee, WI) using a circular, 8-channel head coil. BOLD images covering the entire brain were obtained along the axial plane using an echo-planar imaging (EPI) sequence (TR/TE = 3000/60 ms; 32 slices acquired in sequential order, slice thickness = 4 mm; field of view = 25.6 cm; flip angle = 90°; matrix size = 64 × 64). During the experimental task, subjects performed a total of 8 blocks, each block lasts 21 seconds, and four blocks were presented for each condition. The conditions were alternated, and the total run length was 6 : 12 minutes. A total of 124 brain volumes were obtained. For reasons of image acquisition time and experimental design, 12 brain volumes per task were discarded, leaving 112 volumes for posterior analysis. The discarded volumes were the first two, which corresponded to messages given to prepare the subjects to begin the task. Then, before each activation block, one volume that corresponded to a task instruction reminder and served as a cue to begin the task was eliminated (8 volumes). Finally, the last two volumes were eliminated because they were messages indicating that the task had been completed.

### 2.3. Data Analyses

Demographic and behavioral results were analysed using SPSS (IBM Corporation, released 2011). An analysis of variance (ANOVA) was done to assess the main and interaction effects of task condition and disease status on cognitive performance. This analysis was conducted using group (patients and controls) as a between-group factor and the two conditions (A and B) as the within-subject factor, with the percentage of correct answers and simple reaction times as the dependent variables.

### 2.4. Image Processing

fMRI analyses were carried out using the SPM8 computer package (http://www.fil.ion.ucl.ac.uk/spm/software/spm8/). Prestatistical processing consisted of motion correction, readjustment to voxel size, and normalization according to the MNI (Montreal Neurological Institute) reference. For smoothening, a Kernel Gaussian filter three times the voxel size was used on the *x*-, *y*-, and *z*-axes.

Brain activations in response to the two conditions were examined by performing a first-level general linear model (GLM) analysis for each subject using a statistical threshold of *α* = .05. To compare activation patterns between groups and conditions, a second-level GLM analysis was conducted using the same statistical threshold and applying posterior correction with a Bonferroni procedure to reduce nominal type I error.

## 3. Results

### 3.1. Behavioral Performance

The analysis of the behavioral results while performing the experimental tasks considered only correct responses and reaction times, since the incorrect response and omission percentages were too small to add insight to the results. In general, patients and controls showed similar behavioral performances (Table 2). Interestingly, both groups seemed to perform worse when evaluating condition A (direct order), though this difference did not reach statistical significance (Table 3).

Patients and controls did not differ in their accuracy (*F*(1, 30) = 0.075; *p* = .786) or reaction times in either condition (*F*(1, 30) = 0.011; *p* = .919), but when the within-subject differences between conditions were compared for the two groups, the number of correct responses differed significantly (*F*(1, 30) = 4.35; *p* = .046). No relevant between-group differences were found for reaction times (*F*(1, 30) = 0.073; *p* = .789), probably because responses were purposely delayed. No significant interactions were observed between groups and conditions for correct responses (*F*(1, 30) = 0.484; *p* = .492) or reaction times (*F*(1, 30) = 0.084; *p* = .775).

### 3.2. Imaging Results

**Figure 2:**
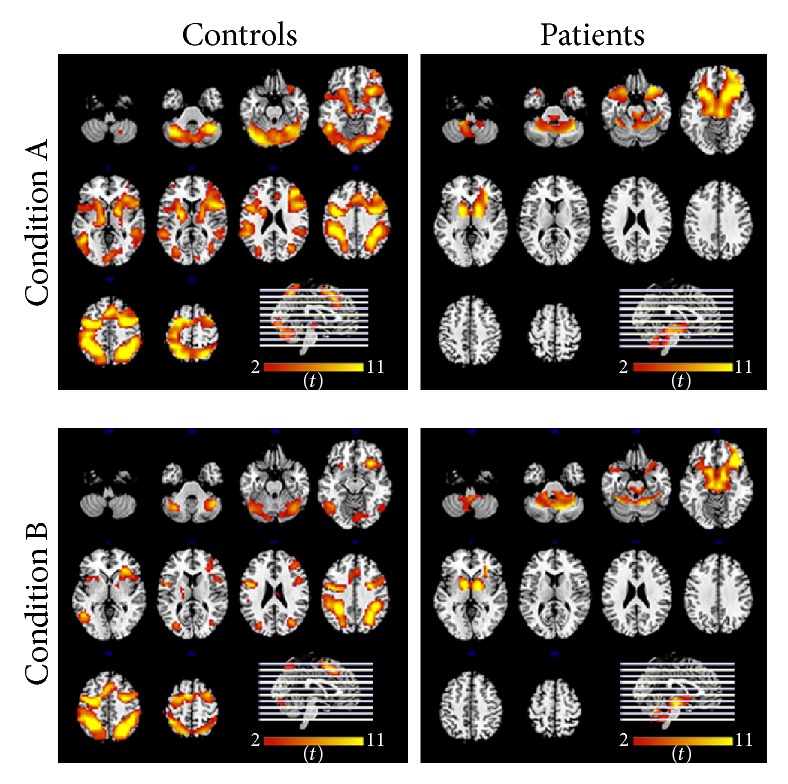
Statistical parametric maps of regions of greatest activation for each condition for both groups.

In the first-level analyses of each subject, patients and controls showed similar activations in the bilateral parietal lobe, premotor cortex, superior frontal gyrus, and cerebellum. However, the second-level analyses showed that cluster size and activation intensity differed between groups. Brain activations in response to each condition of the visuospatial working memory task for the two groups are shown in Figure 2. The regions of greatest BOLD activation in the control group for condition A were located in the right superior and inferior parietal lobes, left premotor cortex, left superior frontal gyrus, right inferior frontal cortex, bilateral cerebellum (tonsil and pyramid), and left putamen. In the patients group, however, the main brain activations while performing condition A appeared in the right inferior and medial frontal cortex, bilateral cerebellum (i.e., parts of the tonsil, tuber, declive, pyramid, and semilunar lobule), and left putamen. The control group showed activations related to condition B in the right medial and superior frontal gyrus, bilateral superior and inferior parietal lobes, left premotor cortex, and bilateral cerebellum (tonsil and pyramid), while the patients showed cerebral activations primarily in the right inferior frontal gyrus, bilateral cerebellum (right tonsil, left tuber, and pyramid), the right putamen, medial globus pallidus, and substantia nigra in the midbrain.

The functional activation pattern in controls was similar in both conditions but, in contrast, patients showed fewer activated clusters which were smaller than those found in controls (Table 4). Healthy controls showed greater activation clusters in the right superior frontal gyrus and bilateral parietal lobe that did not survive the statistical threshold in the patient group. Furthermore, T1D patients showed greater activations in the right inferior frontal gyrus, cerebellum, basal ganglia, and substantia nigra.

Given that condition B represented a higher working memory load level than condition A, we were particularly interested in the second-level analysis using a unilateral contrast to compare the two conditions.  Table 5 shows three main cluster activations in the control group, while no activation clusters survived the statistical threshold in the patient group. The brain activations observed in the control group were similar to those found in previous analyses, thus confirming the notion that there were fewer activated regions in patients and that they had a lower activation magnitude.

## 4. Discussion

The present study demonstrates that patients with T1D show a different brain activation pattern than healthy control subjects although, as expected, there were no statistically-significant differences in their behavioral performance. The lack of behavioral differences might be due to the fact that our patients could be characterized as having a higher cognitive profile framework. This notion seems to be supported by their normal IQ, their age-appropriate educational level (above the population average), and normal social adaptability, since all were studying or working at the time of the study. Furthermore, all patients were recruited through hospital services where they were regular patients who had monthly monitoring visits. Therefore, we can assume that their adherence to treatment was appropriate.

The finding that both groups showed more accurate responses in condition B than condition A was actually unexpected. However, subjects were instructed and trained on the tasks prior to performance, and this might have influenced their cognitive strategies such that greater effort was allocated to solving the more difficult condition (B), probably leading to an increase in task-performance efficiency.

All of the brain regions usually described as being involved in visuospatial working memory processing [32] were activated in both patients and controls during this study. However, results show that the T1D patients showed a different functional brain activation pattern, one that presented fewer activation clusters in the parietal lobe, premotor cortex, and superior frontal gyrus, compared to the healthy controls. Indeed, the control group showed activation clusters located in the inferior frontal gyrus, basal ganglia, and cerebellum, though those activations were greater in patients. Moreover, patients showed an important activation cluster in the substantia nigra of the midbrain. Briefly, then, patients showed several subcortical activations in addition to the cortical activations usually required for processing in working memory.

These results are in line with those of Wessels et al. [34], which suggest that if a pathological process alters the response in certain region, this will also affect the activation in other regions to compensate for functional loss. In the present experiment, the integrity of inferior prefrontal cortex, basal ganglia, and cerebellum would be intact, while dorsolateral prefrontal cortex and parietal cortex could be affected due to a decreased metabolism. However, this finding could represent the opposite; that is, inferior prefrontal cortex, basal ganglia, and cerebellum could be affected. An explanation for this alternative is the possibility that these brain regions would have a decreased resting state deactivation and remain “active” to maintain task performance or to compensate for functional loss as it was proposed by Wessels and colleagues [34]. In our group of young patients, it seems a successful mechanism, but it could eventually fail with aging and/or the presence of disease complications.

Evidence points towards the contribution of the inferior prefrontal cortex, basal ganglia, and cerebellum to the enhancement of working memory processing. More specifically, the basal ganglia supposedly allow only relevant information into working memory. The globus pallidus is considered the output module of the basal ganglia and is crucial for spatial attention and there is also evidence for involvement of the globus pallidus during working memory-guided movement sequencing [35, 36]. Given that the basal ganglia are high-density dopamine receptor subcortical nuclei, they are also involved during the selective updating of working memory via a dopaminergic mesostriatocortical network [37, 38]. Therefore, one could assume that activation of this network may be a compensatory mechanism employed in an attempt to keep up with the updating process. According to this assumption, an analysis that split high- and low-performing subjects on a working memory task [38] reported that individuals who have difficulty with selective updating engage the mesostriatocortical loop to a greater extent as a compensatory mechanism to help update the new information in working memory. On the other hand, this study found that parietal activations that the authors suggested may have contributed to the attention processes required to selectively update working memory content. Thus, we might expect that brain regions such as the parietal lobes play a significant role in these processes, particularly while processing the current experimental task. However, patients in the present study did not show these parietal activations. In line with the previous argument, it is possible that in the group of patients the basal ganglia and the cerebellum may be engaged as part of a mechanism to compensate for decreased parietal lobe activations.

In this regard, some studies suggest that the cerebellum could be playing a gatekeeper role by controlling incoming working memory information due to its well-known involvement in attentional processes [39]. These studies further propose that the cerebellum might modulate filtering processes in the basal ganglia via corticocerebellar circuitry. Alternatively, there is strong evidence supporting the idea that the cerebellum may operate as a kind of internal timing system by providing some form of temporal knowledge on various cognitive tasks [40], such as sequencing stimuli [41]. Therefore, the cerebellum might contribute to the encoding of the sequence of stimuli in our task, which is relevant to working memory.

However, both the corticocerebellar circuitry and the dopaminergic mesostriatocortical network seem to point to filtering and selective updating processes for working memory, and the prefrontal dorsolateral cortex would be the cortical connection in these networks. Moreover, we expected strong activations of this region, given its important role in monitoring and manipulation processes in working memory. Conversely, patients strongly activated the right inferior frontal cortex and, though to a lesser extent, other frontal areas. A study using voxelwise lesion-behaviour brain mapping in stroke patients while performing a visuospatial working memory task found that the right prefrontal cortex is crucial for actively maintaining relevant information online [36]. In this regard, a recent study [42] proposed that the right inferior frontal cortex and the fronto-basal-ganglia networks implement inhibitory control in the form of a brake. In the present study, subjects were instructed to delay their responses until a specific written command appeared on the screen; thus right inferior frontal cortex activation in patients might be interpreted as reflecting this inhibitory braking effect, probably magnified by a slower metabolic disengagement of this structure due to inefficient insulin mobilization. Alternatively, it might reflect an adaptive mechanism that seeks to actively maintain information in working memory in order to compensate its less efficient handling.

In recent years, research has focused on the idea that metabolic disturbances in the structure and function of the central nervous system caused by T1D are mainly due to hyper- and hypoglycemic extremes. Moreover, it has been reported that chronic glycemic dysregulation could cause a decrease in the scores of neuropsychological tests [2, 27, 31]. However, there are many patients—such as those in the present study—that successfully cope with the illness, while maintaining a functional daily life with no evidence of cognitive impairment. Indeed, other studies have found similar behavioral performance between T1D patients and healthy controls [8, 23] during working memory task performance. Those studies also reported distinct brain activation patterns in the different groups. But those differences were observed during a specific glycometabolic state (e.g., hypoglycemia) or included patients with illness complications such as retinopathy. Nevertheless, the authors suggest that brain regions (e.g., right superior frontal gyrus, parietal cortex, and cerebellum) that showed increased activation may have been recruited to help preserve cognitive performance in order to compensate for cerebral inefficiency attributed to reduced brain resources. They further suggested that this occurred by maintaining activity from euglycemia to hypoglycemia in task-relevant regions and by failing to suppress activation in the default mode network.

Moreover, a recent study reported structural differences in the gray and white matter between a group of very young children with early-onset T1D and a control group. Those differences involved the lateral frontal, medial frontal, occipital, and cerebellar brain regions [2]. According to the authors, these regions may be particularly vulnerable to the glycemic effects of early-onset TID. In light of the present results, we must consider that brain vulnerability could extend throughout neurodevelopment and that the deleterious effects might involve not only the structure but also the functioning of the brain. In this regard, the effect of nonclinical microvascular disease due to chronic hyperglycemia might be playing a role as a potential determinant [34]. However, several studies in T1D patients with a long history of disease and cognitive alterations have reported that these patients showed an improvement in neuropsychological test performance after treatment with either islets or pancreas transplantation due to the achievement of sustained normoglycemia [15, 16]. These relevant findings reinforce the crucial role of neural plasticity in the potential reversibility of brain alterations caused by T1D.

There are several limitations regarding this study. Our main limitation is that we initially assumed that the glucose levels of the control group would be normal due to the lack of clinical symptoms of diabetes or other disease that could affect them. Therefore, we did not measure glucose levels in the control group. On the other hand, despite our small sample size, in the first level analyses made by subject individually, we found regular effects across all subjects. We did not find any kind of outliers or alterations in brain functional patterns of activations that might lead to different individual contributions to the changes observed between groups.

Nonetheless, our results suggest that early influences during neurodevelopment could lead to functional adaptive changes as a means of maintaining cognitive adaptability. With respect to the educational achievements of our group of patients, we can speculate that they have been successful due to these adaptive changes in their neural networks. This suggests the possibility that intrinsic neural network differences might develop to support task demands in T1D, though further studies using neuroimaging techniques such as brain connectivity methods will be necessary to better evaluate this hypothesis.

## Figures and Tables

**Table 1:** Demographic and clinical characteristics of the participants.

	T1D patients	Healthy controls
*n*	16	16
Age (years)	20.6 (4.0)	21.13 (4.41)
Sex (men/women)	9/7	9/7
Education (years)	12.69 (2.87)	13.31 (2.75)
Intelligence quotient	103.88 (7.40)	113.06 (7.30)
Diabetes duration (years)	10.44 (5.37)	—
HbA_1c_ (%)	8.91 (2.09)	—
HbA_1c_ (mmol/mol)	74 (22.8)	—
Last fasting plasma glucose (mg/dL)	128.54 (60.05)	—

Data are means (SD). *n* = number of cases, HbA_1c_ = glycated hemoglobin, and *p* value = statistical significance.

**Table 2:** Descriptive statistical results of task performance.

	Group	Condition	
		A	B
Correct responses (%)	Controls	88.28 (8.50)	94.53 (11.15)
	Patients	89.06 (10.07)	92.19 (11.06)

Reaction times (ms)	Controls	589.68 (183.64)	590.00 (149.84)
	Patients	600.19 (143.31)	590.70 (164.24)

Data are mean (SD).

**Table 3:** Summary results of ANOVA for behavioral results.

		*F*	df	*p*	*η* ^2^	1 − *β*
Correct responses	Condition	4.35	1, 30	.046	.127	.524
	Group	0.075	1, 30	.786	.003	.058
	Condition *∗* group	0.484	1, 30	.492	.016	.103

Reaction times	Condition	0.073	1, 30	.789	.002	.058
	Group	0.011	1, 30	.919	.000	.051
	Condition *∗* group	0.084	1, 30	.775	.003	.059

*F* = Snedecor's *F* statistic; *p* = statistical significance; df = degrees of freedom; *η* ^2^ = effect size.

**Table 4:** Statistically significant activation clusters for both groups in each condition.

Group/condition	Brain region	BA	H	Cluster	*Z*max	MNI coordinates		
						*x*	*y*	*z*
Healthy controls								
A	Superior parietal lobe; superior frontal gyrus	7	R	17,760	6.59	30	−60	50
B	Precentral gyrus; superior parietal lobe	6	L	14,495	6.74	−34	−8	50

T1D patients								
A	Inferior frontal gyrus; putamen; medial frontal gyrus	47	R	2,281	5.83	34	20	−14
B	Putamen; inferior frontal gyrus	47	R	1,620	5.30	14	0	−2
	Superior frontal gyrus	11	L	8	3.01	−30	48	−18

BA = Brodmann's area related to cluster peak activation, H = hemisphere, R = right, L = left, *Z*max = maximum *Z* score of the main cluster activation, and MNI = Montreal Neurological Institute three axis coordinates (*x*, *y*, and *z*).

**Table 5:** Statistically significant activation clusters for condition A versus condition B unilateral contrast.

Group	Brain region	BA	H	Cluster	*Z*max	MNI coordinates		
						*x*	*y*	*z*
Healthy controls	Parahippocampal gyrus; medial frontal gyrus	30	L	82	3.41	−18	−40	10
	Superior parietal lobe; inferior parietal lobe;	7	L	112	2.56	−22	−64	46
	Caudate	—	R	41	2.51	26	−36	18

T1D patients	No statistical significant activation clusters found^*∗*^							

BA = Brodmann's area related to cluster peak activation, H = hemisphere, R = right, L = left, *Z*max = maximum *Z* score of the main cluster activation, and MNI = Montreal Neurological Institute three axis coordinates (*x*, *y*, and *z*).

^*∗*^ *p* > .05.
